# Supplementary material for: Molecular Pathogenesis of the Coronin Family: CORO2A Facilitates Migration and Invasion Abilities in Oral Squamous Cell Carcinoma
Source: Int J Mol Sci. 2021 Nov 24;22(23):12684. doi: 10.3390/ijms222312684 (PMC8657730; doi:10.3390/ijms222312684)
Supplement: Supplementary file 1 [file ijms-22-12684-s001.zip › IJMS CORO2A facilitates migration and invasion abilities_ in OSCC supplementary figures.pptx]

## Slide 1
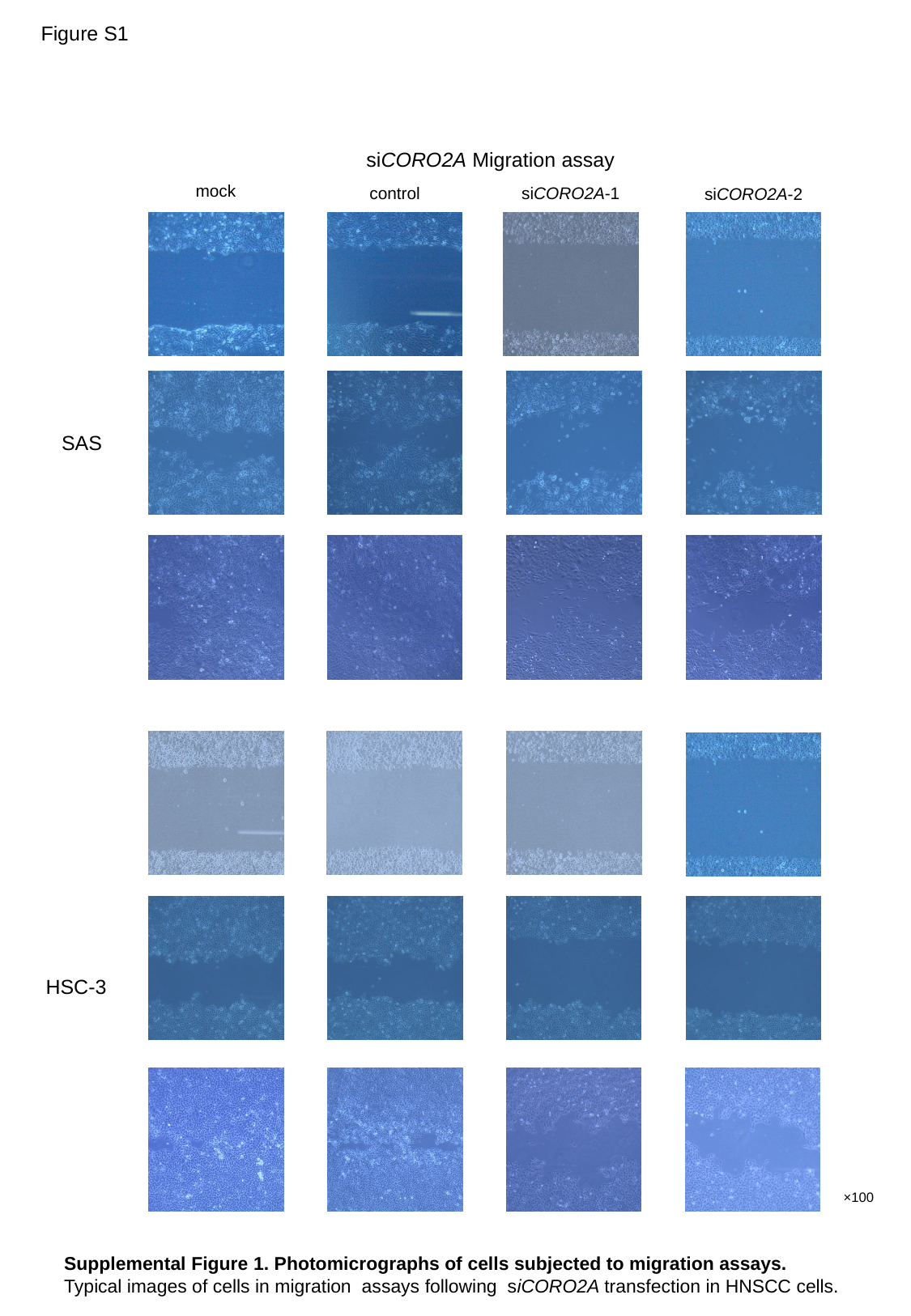

Figure S1
siCORO2A Migration assay
mock
control
siCORO2A-1
siCORO2A-2
SAS
HSC-3
×100
Supplemental Figure 1. Photomicrographs of cells subjected to migration assays.
Typical images of cells in migration assays following siCORO2A transfection in HNSCC cells.

## Slide 2
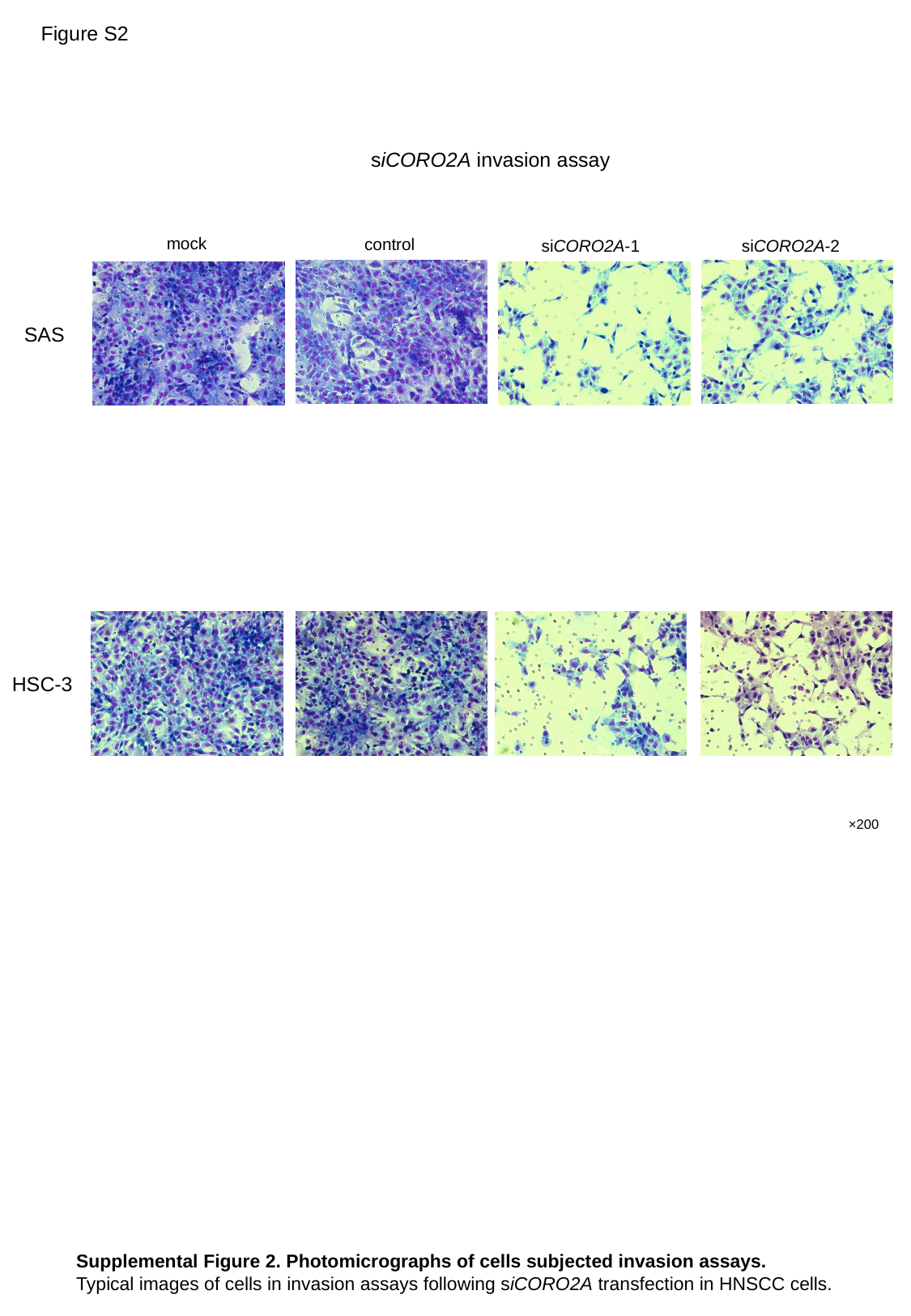

Figure S2
siCORO2A invasion assay
mock
control
siCORO2A-1
siCORO2A-2
SAS
HSC-3
×200
Supplemental Figure 2. Photomicrographs of cells subjected invasion assays.
Typical images of cells in invasion assays following siCORO2A transfection in HNSCC cells.

## Slide 3
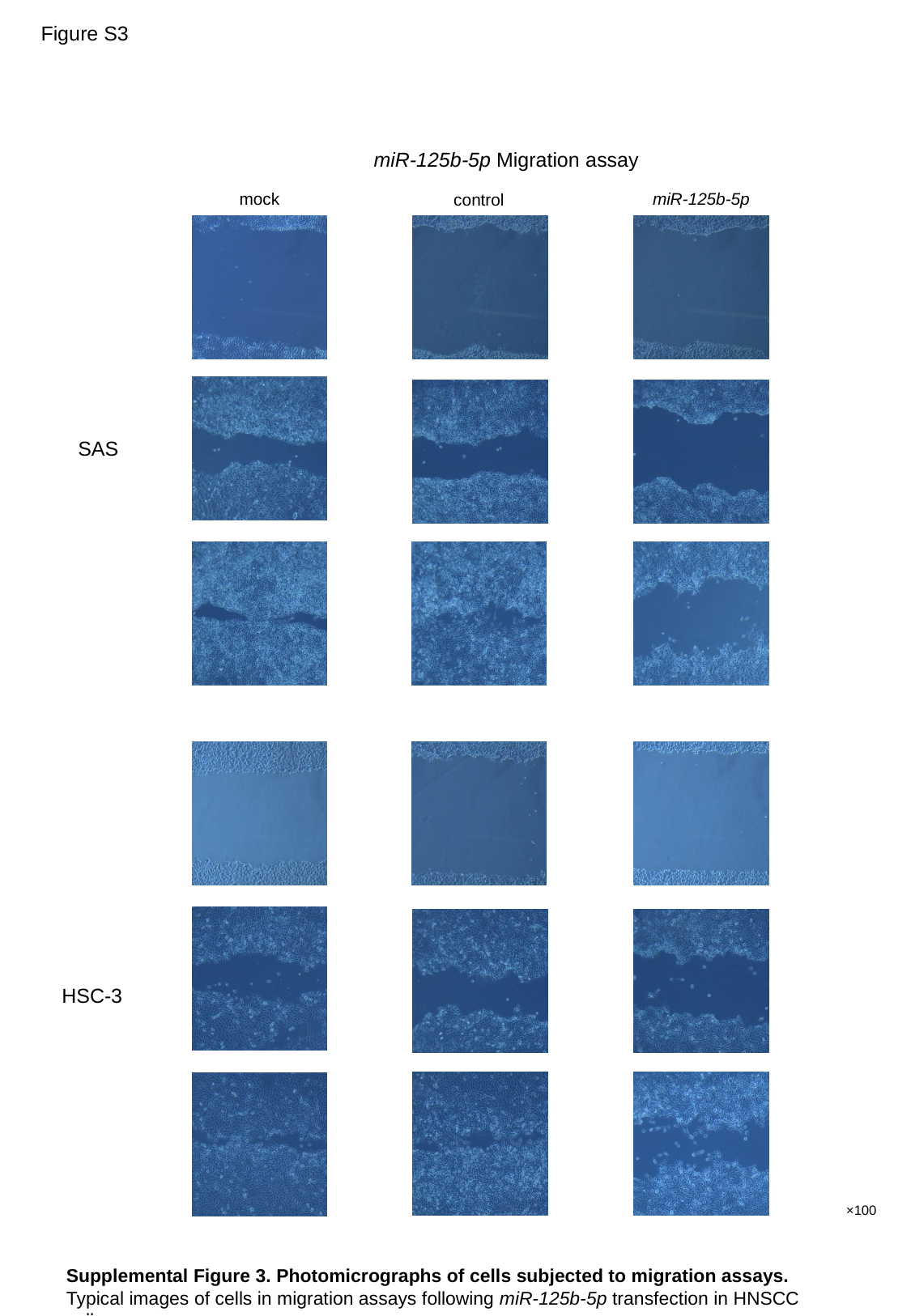

Figure S3
miR-125b-5p Migration assay
mock
miR-125b-5p
control
SAS
HSC-3
×100
Supplemental Figure 3. Photomicrographs of cells subjected to migration assays. Typical images of cells in migration assays following miR-125b-5p transfection in HNSCC cells.

## Slide 4
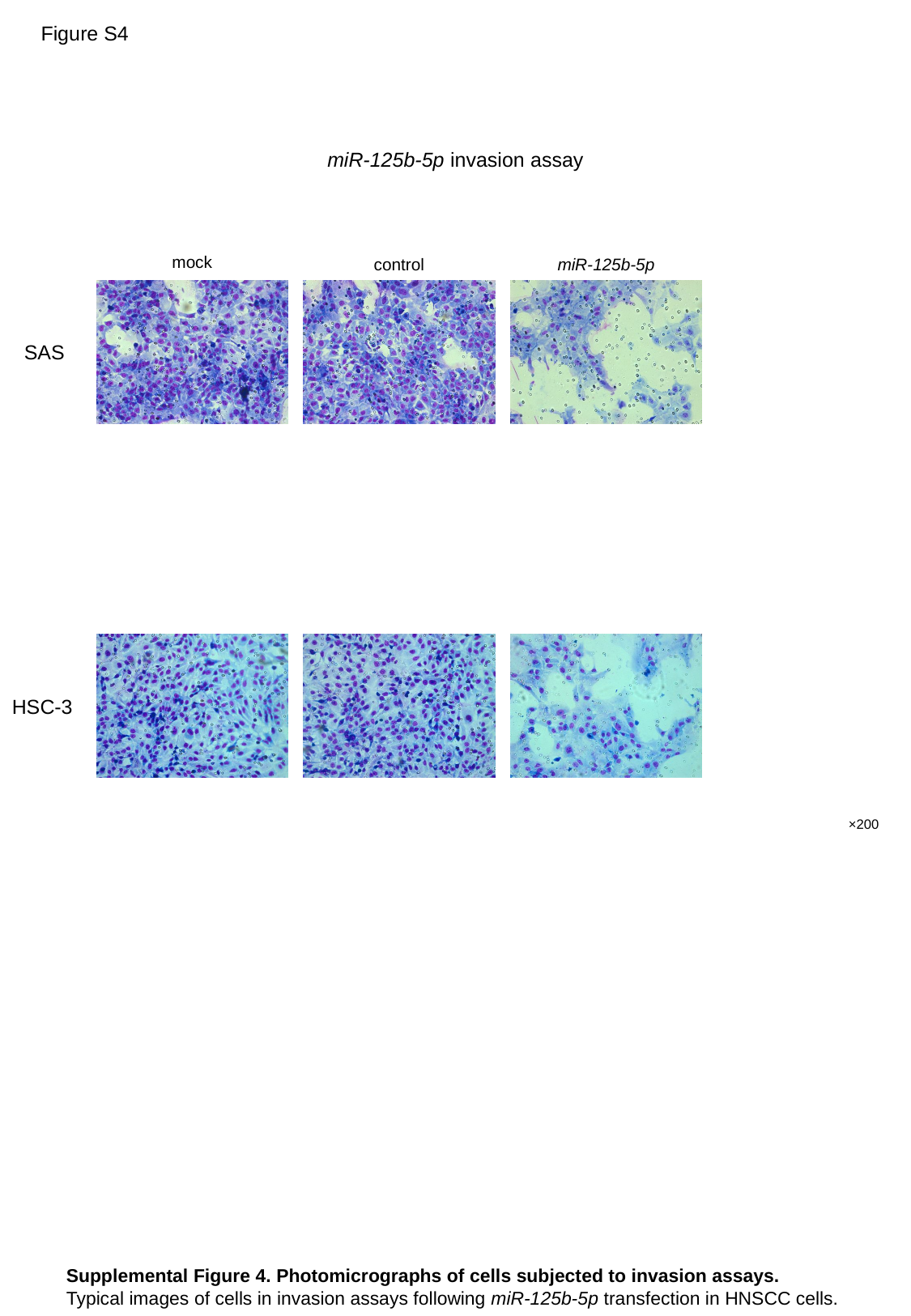

Figure S4
miR-125b-5p invasion assay
mock
miR-125b-5p
control
SAS
HSC-3
×200
Supplemental Figure 4. Photomicrographs of cells subjected to invasion assays.
Typical images of cells in invasion assays following miR-125b-5p transfection in HNSCC cells.

## Slide 5
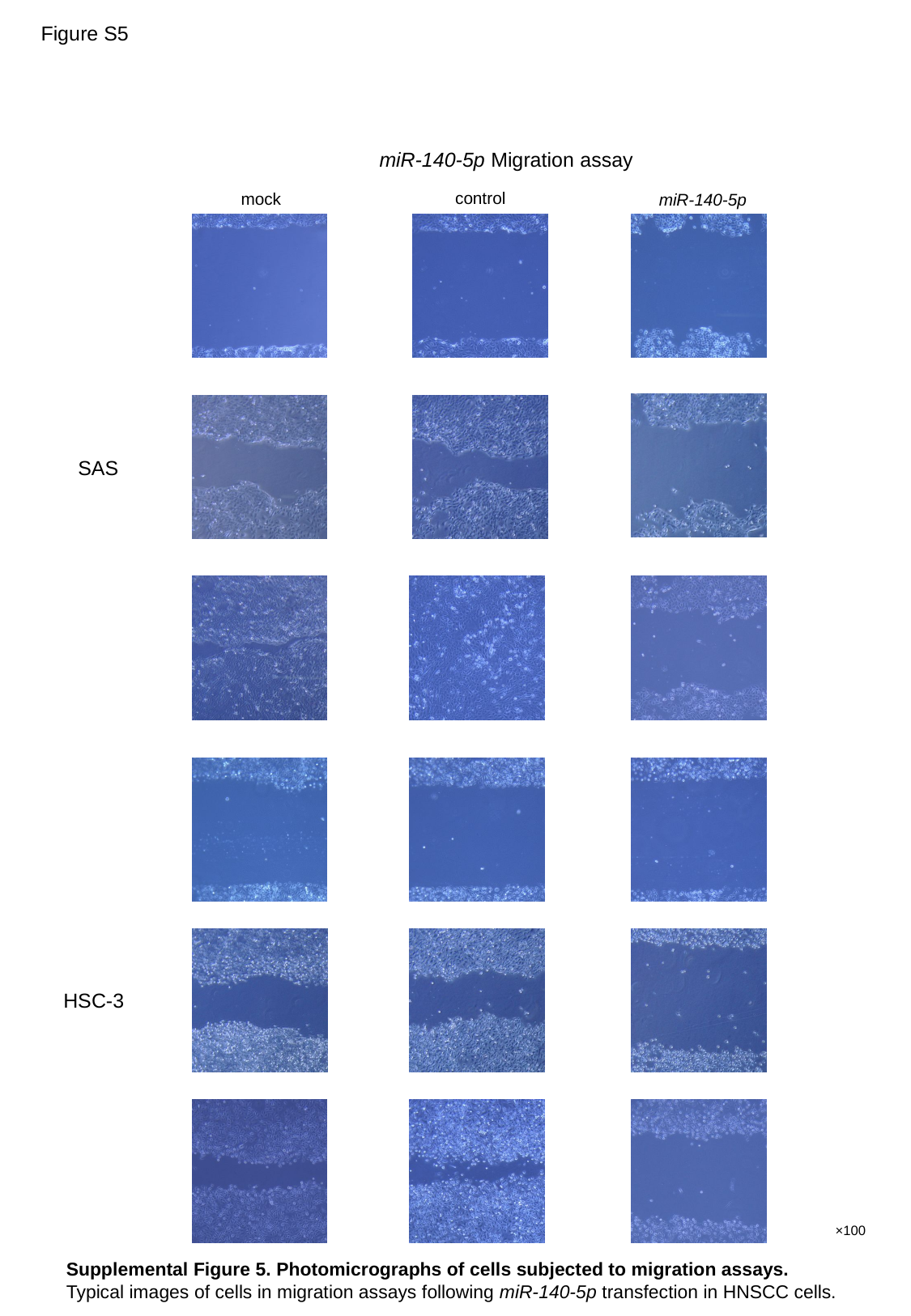

Figure S5
miR-140-5p Migration assay
control
mock
miR-140-5p
SAS
HSC-3
×100
Supplemental Figure 5. Photomicrographs of cells subjected to migration assays. Typical images of cells in migration assays following miR-140-5p transfection in HNSCC cells.

## Slide 6
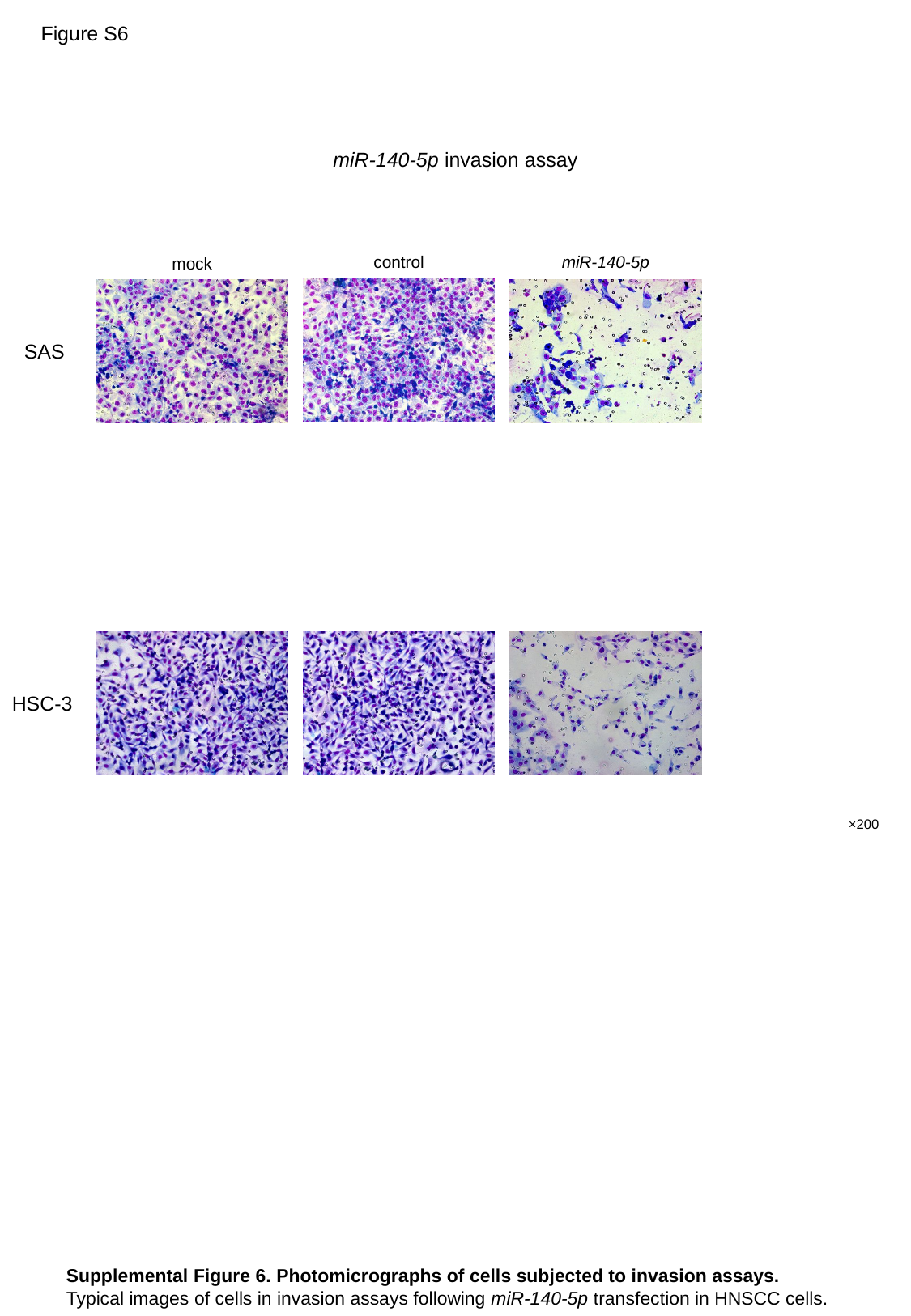

Figure S6
miR-140-5p invasion assay
miR-140-5p
control
mock
SAS
HSC-3
×200
Supplemental Figure 6. Photomicrographs of cells subjected to invasion assays.
Typical images of cells in invasion assays following miR-140-5p transfection in HNSCC cells.

## Slide 7
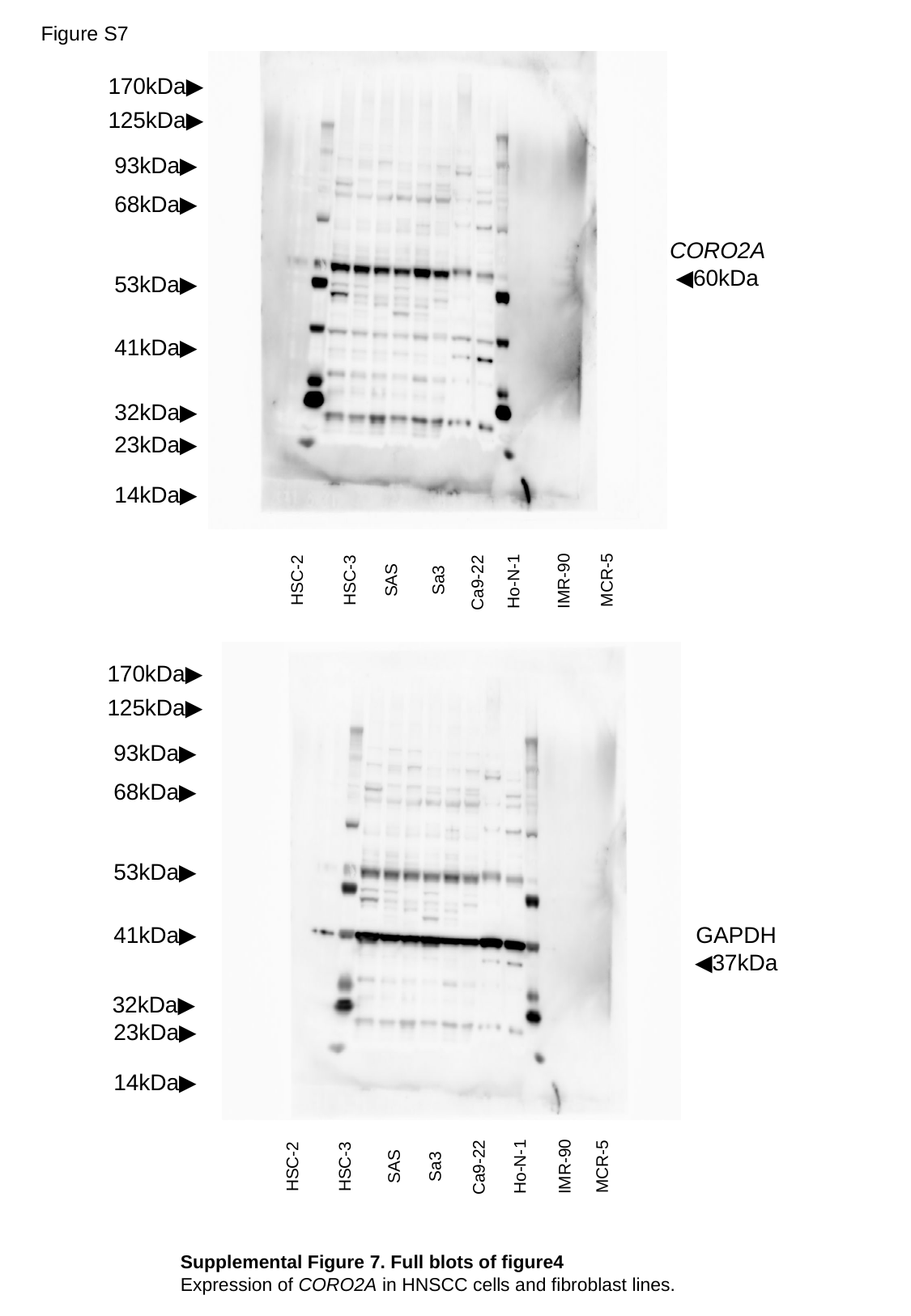

Figure S7
170kDa▶
125kDa▶
93kDa▶
68kDa▶
CORO2A ◀60kDa
53kDa▶
41kDa▶
32kDa▶
23kDa▶
14kDa▶
Sa3
SAS
HSC-2
HSC-3
MCR-5
IMR-90
Ho-N-1
Ca9-22
170kDa▶
125kDa▶
93kDa▶
68kDa▶
53kDa▶
41kDa▶
GAPDH ◀37kDa
32kDa▶
23kDa▶
14kDa▶
Ho-N-1
Sa3
HSC-2
HSC-3
SAS
IMR-90
MCR-5
Ca9-22
Supplemental Figure 7. Full blots of figure4
Expression of CORO2A in HNSCC cells and fibroblast lines.

## Slide 8
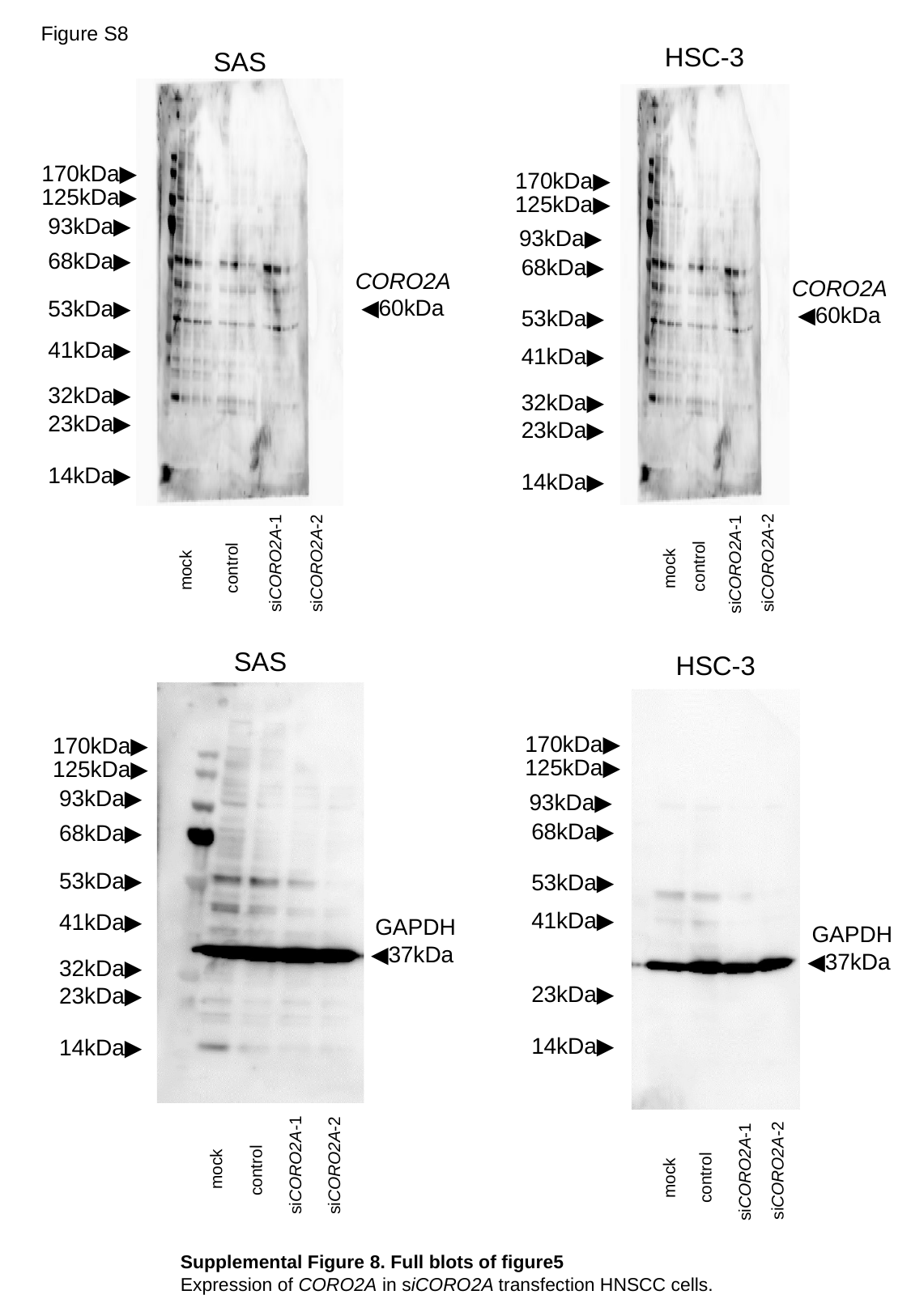

Figure S8
HSC-3
SAS
170kDa▶
170kDa▶
125kDa▶
125kDa▶
93kDa▶
93kDa▶
68kDa▶
68kDa▶
CORO2A ◀60kDa
CORO2A ◀60kDa
53kDa▶
53kDa▶
41kDa▶
41kDa▶
32kDa▶
32kDa▶
23kDa▶
23kDa▶
14kDa▶
14kDa▶
siCORO2A-2
siCORO2A-1
siCORO2A-2
siCORO2A-1
control
control
mock
mock
SAS
HSC-3
170kDa▶
170kDa▶
125kDa▶
125kDa▶
93kDa▶
93kDa▶
68kDa▶
68kDa▶
53kDa▶
53kDa▶
41kDa▶
41kDa▶
GAPDH
◀37kDa
GAPDH
◀37kDa
32kDa▶
23kDa▶
23kDa▶
14kDa▶
14kDa▶
siCORO2A-1
siCORO2A-2
mock
siCORO2A-2
control
siCORO2A-1
mock
control
Supplemental Figure 8. Full blots of figure5
Expression of CORO2A in siCORO2A transfection HNSCC cells.

## Slide 9
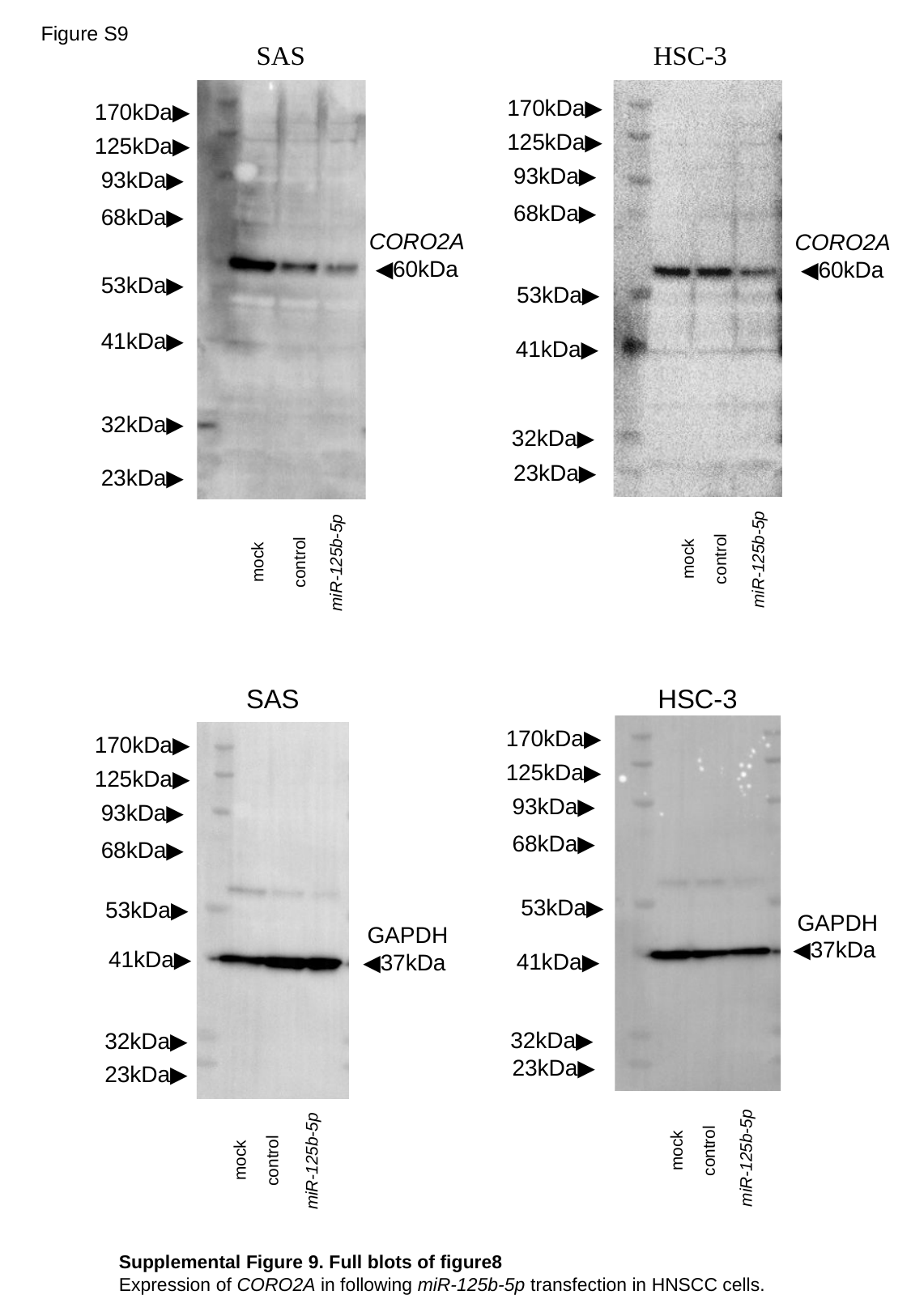

Figure S9
SAS
HSC-3
170kDa▶
170kDa▶
125kDa▶
125kDa▶
93kDa▶
93kDa▶
68kDa▶
68kDa▶
CORO2A ◀60kDa
CORO2A ◀60kDa
53kDa▶
53kDa▶
41kDa▶
41kDa▶
32kDa▶
32kDa▶
23kDa▶
23kDa▶
mock
control
miR-125b-5p
mock
control
miR-125b-5p
SAS
HSC-3
170kDa▶
170kDa▶
125kDa▶
125kDa▶
93kDa▶
93kDa▶
68kDa▶
68kDa▶
53kDa▶
53kDa▶
GAPDH
◀37kDa
GAPDH
◀37kDa
41kDa▶
41kDa▶
32kDa▶
32kDa▶
23kDa▶
23kDa▶
mock
control
miR-125b-5p
mock
control
miR-125b-5p
Supplemental Figure 9. Full blots of figure8
Expression of CORO2A in following miR-125b-5p transfection in HNSCC cells.

## Slide 10
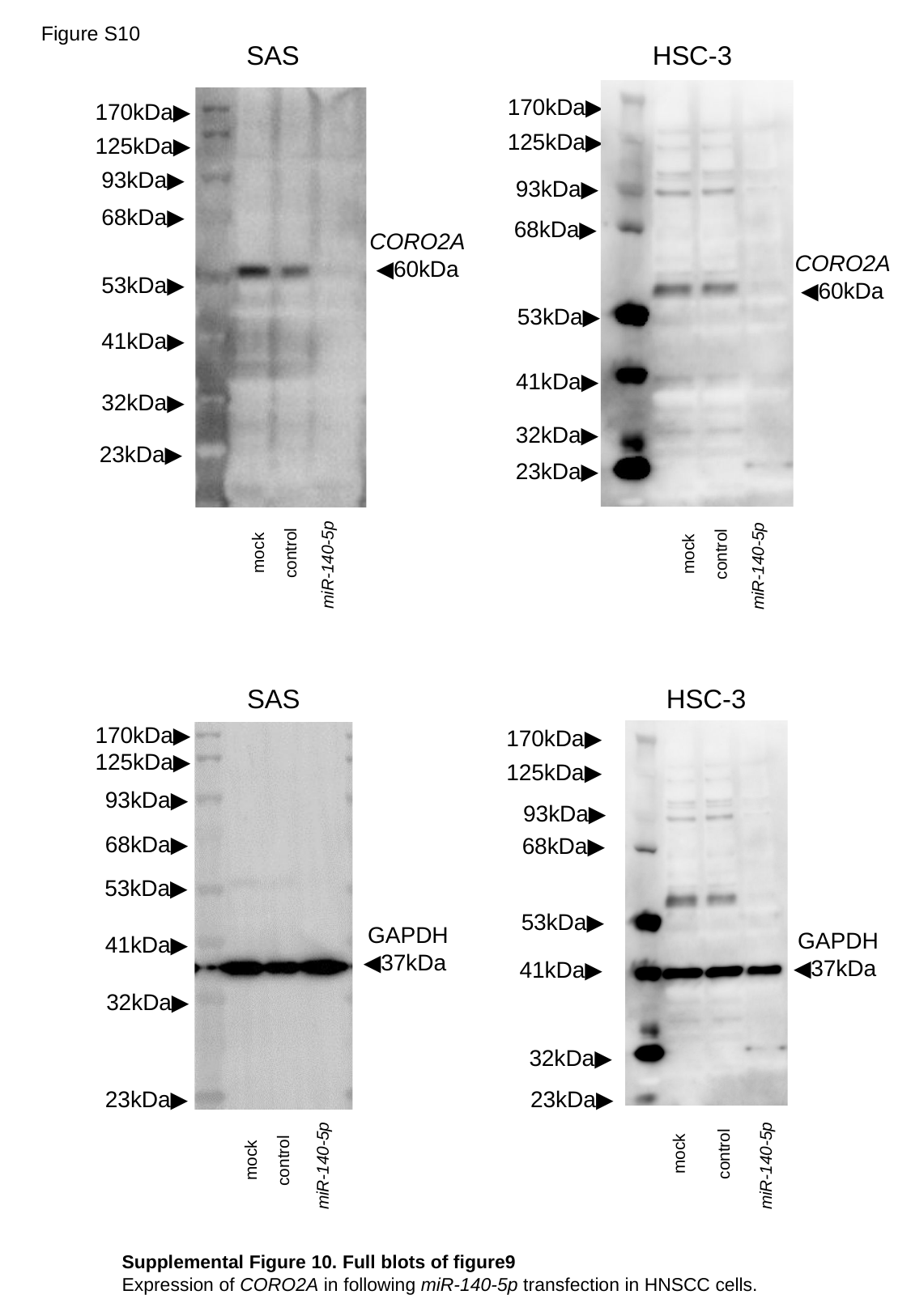

Figure S10
SAS
HSC-3
170kDa▶
170kDa▶
125kDa▶
125kDa▶
93kDa▶
93kDa▶
68kDa▶
68kDa▶
CORO2A ◀60kDa
CORO2A ◀60kDa
53kDa▶
53kDa▶
41kDa▶
41kDa▶
32kDa▶
32kDa▶
23kDa▶
23kDa▶
mock
control
miR-140-5p
mock
control
miR-140-5p
SAS
HSC-3
170kDa▶
170kDa▶
125kDa▶
125kDa▶
93kDa▶
93kDa▶
68kDa▶
68kDa▶
53kDa▶
53kDa▶
GAPDH
◀37kDa
GAPDH
◀37kDa
41kDa▶
41kDa▶
32kDa▶
32kDa▶
23kDa▶
23kDa▶
mock
control
miR-140-5p
mock
control
miR-140-5p
Supplemental Figure 10. Full blots of figure9
Expression of CORO2A in following miR-140-5p transfection in HNSCC cells.
